# Hereditary Hemochromatosis and Polydactyly: A Case Report

**DOI:** 10.7759/cureus.84830

**Published:** 2025-05-26

**Authors:** Shloka Reddy, Steve Thomas

**Affiliations:** 1 General Medicine, Sri Ramachandra Institute of Higher Education and Research, Chennai, IND; 2 Hematology, Sri Ramachandra Institute of Higher Education and Research, Chennai, IND

**Keywords:** genetic counseling, hfe, iron intake, joint pains, polydactyly

## Abstract

Hereditary hemochromatosis is a genetic disorder caused by mutations in genes regulating hepcidin, leading to increased iron absorption and progressive accumulation in the body. It is commonly encountered in the Western population, but prevalence in the Indian population, especially of the *H63D* homozygous mutation variant, is on the lower end. Herein is a case of a young male with a paternal hereditary trait of polydactyly, on regular follow-up for anti-hypertensives, who presented with fatigue and multiple large joint pains. Unexplained fatigue, along with a significant family history, warranted further evaluation. Serum iron levels, transferrin saturation, and ferritin levels were elevated. Hence, the patient was referred to the hematology department, for which *HFE *genetic mutation analysis was done, and* a H63D* homozygous mutant was noted. Target end-organ damage was assessed. The patient was monitored and advised to reduce iron intake. Genetic counseling was also done, along with screening of first- and second-degree relatives.

## Introduction

Hereditary hemochromatosis is an autosomal recessive disorder with low penetrance. It is characterized by increased iron absorption, which leads to total body iron overload. The incidence of an abnormality of increased iron absorption was historically found in the 1980s and 1990s, following the discovery of the *HFE* gene in 1996. Population screening has found the incidence of 6.4% in White Americans as well as 4-5% in White Europeans and is extremely rare in East Asia (<0.1%). In East Asia, variants in genes other than *HFE*, such as *HJV*, *HAMP*, and *TFR2,* together known as non-*HFE* hemochromatosis, are more prevalent [1]. *HFE* gene homozygosity takes several decades of excess iron absorption without co-existing blood loss to be clinically significant [2]. Incidence of homozygous *C282Y* is more than *H63D*, but genetic or environmental modifiers, such as blood transfusion and dietary iron intake, also contribute. Iron accumulates and causes damage through mechanisms that include oxygen free radicals in the liver, heart, pituitary gland, and pancreas; hence, monitoring is imperative [3]. As per Hemochromatosis and Iron Overload Screening (HEIRS) study, 100,000 individuals were evaluated, out of which >10% were found to be homozygous *C282Y/C282Y*, followed by compound heterozygous *C282Y/H63D*, homozygous *H63D/H63D*, and heterozygous (*C282Y*/wild type or *H63D*/wild type) [4]. Deposition in the liver generally occurs first, followed by other organs, as iron absorbed from the GI tract passes through the liver first. One analysis done at a tertiary referral center over a 10-year period suggested a significant male predominance among individuals with hereditary hemochromatosis [5].

## Case presentation

Presenting history

A young male in his mid-30s, who was a resident of Chennai, India, and a software engineer by profession, with a history of systemic hypertension, being a reformed smoker, and occasional alcohol consumption, presented to our outpatient department for a refill visit. As he entered, at first glance, he appeared to be moderately built and nourished. As a routine check-up, his vitals were checked and recorded as follows: a pulse rate of 86/min and BP of 140/90 mmHg. While going through his history, his regular visits to the orthopedics department in view of complaints of multiple large joint pains sparked concern. The patient had complaints of generalized fatigue.

Examination findings

Examination revealed the evidence of bilateral post-axial polydactyly. On further probing, the patient divulged that it has been a paternal hereditary trait passed on across generations. The patient also mentioned that his father had similar complaints at about the same age, and he also suggested that he had a characteristic skin pigmentation, which led to a lot of stigma. His father was not evaluated for the same. Regarding his family tree, the patient was born of a non-consanguineous marriage; he was the youngest of three siblings, recently married, and planning to have children. With the background of young-onset systemic hypertension, a significant family history, and non-resolving non-specific large joint pains, we further proceeded with a general examination. There were no signs of jaundice, hyperpigmentation, joint tenderness, or subcutaneous nodules. Systemic examination was unremarkable, with no evidence of hepatomegaly.

Diagnostic tests

The patient had brought a file of his recently done blood work-up, complete blood count, renal profile, and liver function tests, all within normal range. The patient had not been on oral iron supplements, with no history of blood transfusion. Considering a paternal hereditary trait of polydactyly, raising suspicion of a coexisting genetic disorder due to a significant family history of skin pigmentation, further work-up done revealed elevated serum iron levels of 178, a transferrin saturation of 53%, and serum ferritin level of 319.80, which prompted a hematology consultation.

*HFE* genetic mutation analysis by the real-time PCR method was done, which detected *H63D* homozygous mutant, whereas *C282Y* and *S65C* gene mutations were not detected. Further target organ damage assessment revealed USG abdomen, which showed grade 1 fatty liver. A peripheral smear done showed normocytic normochromic anemia, with viral markers being non-reactive. A 2D echo test showed normal chamber dimensions and normal LV function. The CT coronary angiography was normal.

Management plan

**Figure 1:**
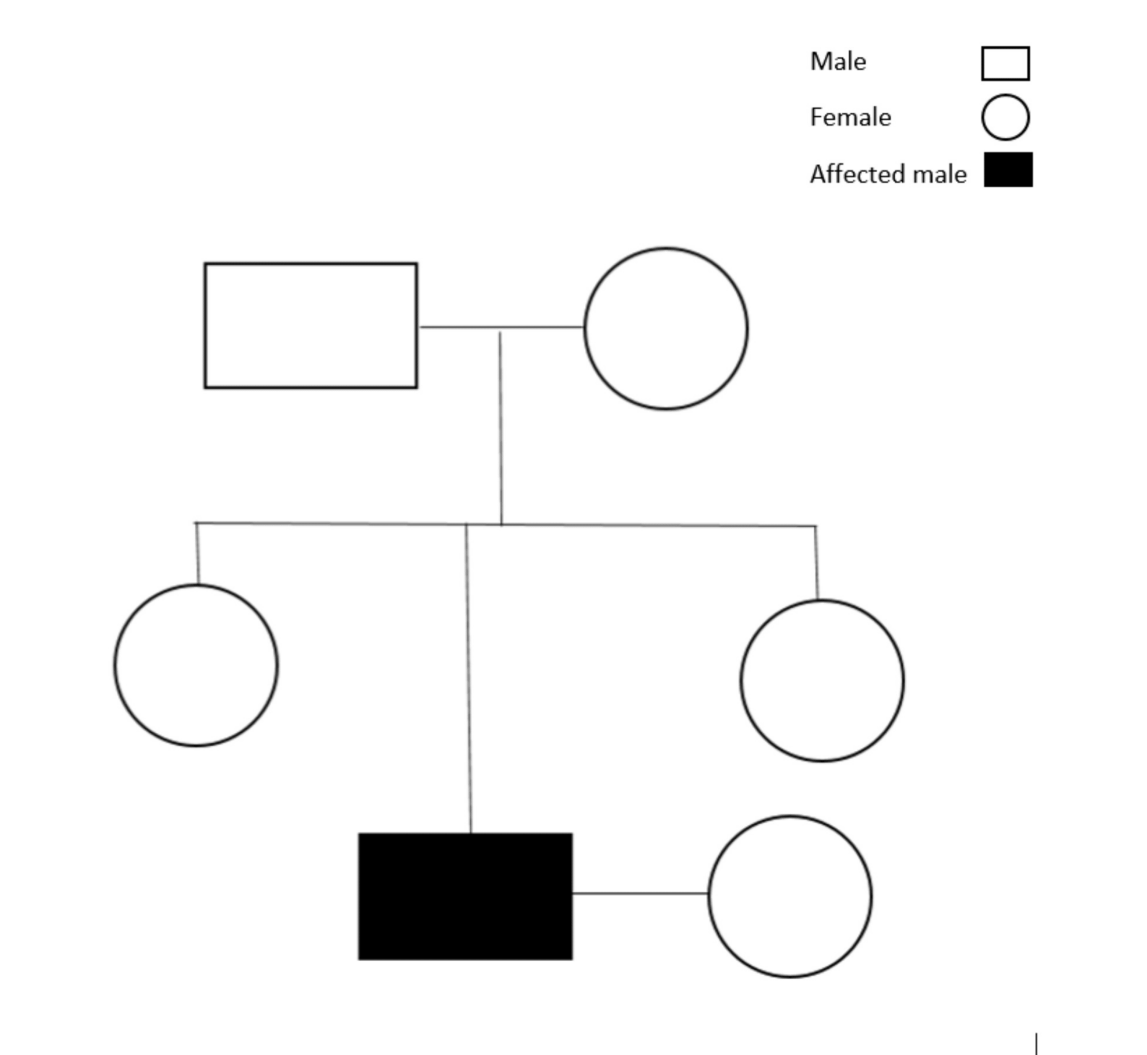
Pedigree analysis Squares represent males, circles represent females, and filled shapes indicate affected individuals. A horizontal line between a square and a circle represents mating. Vertical lines connect parents to offspring.

The patient’s siblings, parents, and partner were advised to undergo genetic mutation analysis. Though the risk of iron overload in first-degree relatives is low with the *H63D* homozygous mutant, genetic testing is imperative. As there is a 25% risk of offspring being affected if his partner is a carrier/affected, the patient was counseled along with his partner regarding the implications (Figure 1). Prenatal testing may be considered if both parents are carriers and there is a strong family history. The patient was advised to wait and watch, and a reduction in iron intake and alcohol consumption was advised. The patient has been on regular follow-ups, and the repeat serum iron level after two months was 126, with transferrin saturation levels of 39, which is clinically asymptomatic (Table 1). Genetic mutation analysis, as suggested for the patient’s first-degree relatives and partner, is awaited.

**Table 1:** Laboratory investigations: first visit and follow-up

Laboratory investigation	Day 1	Day 90	Normal range
Hb	16.5	15.9	13-17 g/dL
MCV	91.6	91	83-101 fL
MCH	31.5	31.2	27-33 pg
ESR	1	7	0-15 mm/h
Serum iron	178	126	75-150 mcg/dL
TIBC	334	323	240-450 mcg/dL
T-saturation	53	39	20-50%
Serum ferritin	319.8	210	25-250 ng/mL
LDH	172	160	100-190 U/L
BUN	19	22	7-18 mg/dL
Serum creatinine	1	1	0.6-1.3 mg/dL
Total bilirubin	1	1.05	0.1-1.2 mg/dL
AST	20	26	0-35 U/L
ALT	27	25	0-41 U/L
ALP	62	59	45-129 U/L
GGT	27	25	0-73 U/L

## Discussion

Epidemiology

In young-onset hypertensives with presenting symptoms of generalized fatigue, arthropathy, and screening for hemochromatosis, especially in populations of European descent, is warranted. In India, it is a rare entity, and genetic screening is not routinely recommended. Although studies on primary hemochromatosis in India are underway, associated genetic mutation detection has been a task. *HFE* gene *C282Y* mutation has not been found except in one family from South India [6]. Case reports on *H63D* mutation analysis in North India reported no significant iron overload, but similar reports on South India statistics are limited [7]. 

Clinical features

Fatigue, especially in the absence of anemia, joint pains, and non-specific symptoms, is commonly seen. Clinical features of iron overload depend on tissue iron amount and contributing factors that can lead to organ dysfunction. The liver, being the principal site of normal iron storage, is the primary site of iron deposition. Cardiac manifestations include dilated cardiomyopathy, diastolic dysfunction, and conduction disturbances. Pancreatic iron overload can lead to type 2 diabetes mellitus, which, combined with skin discoloration, has led to the popular term “bronze diabetes.” Pituitary involvement leads to secondary hypogonadism or hypothyroidism. Other findings include cognitive disturbances and increased susceptibility to infections.

In males, symptoms develop approximately 10 years earlier than females, most likely between 40 and 60 years. In the case discussed, early diagnosis in his mid-30s with no evidence of target end organ damage reduces the risk of life-threatening complications. Hereditary hemochromatosis may be suspected, as in this case, with the presentation of unexplained fatigue, with a probable first- or second-degree relative diagnosed with the same, with high serum ferritin and transferrin saturation (>45%). MRI of the liver and heart, or combined approach, can be used to estimate body iron stores.

Genetic considerations

The presence of post-axial polydactyly with a significant family history raised the possibility of a syndromic presentation. Some genes, like *GLI3*, have been linked to both post-axial polydactyly and hereditary hemochromatosis [8]. Although the *H63D* homozygous mutation has lower penetrance, the patient is at risk for iron overload [9]. This case emphasizes the co-occurrence of two distinct genetic conditions in the same individual and the importance of genetic evaluation.

Management options 

The goal of treatment is to prevent organ damage due to excess iron [10]. Hence, individuals with iron overload are best treated with phlebotomy. Patients without iron overload who have a homozygous mutant can be monitored regularly, as in the case discussed. In the case of heterozygous mutants, other contributing factors must be addressed.

In this case, the patient’s symptoms were non-specific, but timely diagnosis and genetic counseling have reduced the incidence of complications and the need for phlebotomy. It allows for targeted screening evaluation for asymptomatic relatives at risk [11].

## Conclusions

This case highlights the importance of not disregarding any symptom and timely diagnosis of hereditary hemochromatosis, which is a rare entity in India, and regular follow-up will ensure that the patient can live symptom-free without complications. Genetic counseling, especially of first-degree relatives and partners of individuals affected, is imperative. If left untreated, it can lead to early death, including heart failure, cirrhosis, diabetes mellitus, and hepatocellular carcinoma. As reports on hereditary hemochromatosis in South India are limited, this case discussion brings to light the importance of genetic screening tests.

## References

[1] Wallace DF, Subramaniam VN (2016). The global prevalence of HFE and non-HFE hemochromatosis estimated from analysis of next-generation sequencing data. Genet Med.

[2] Hanson EH, Imperatore G, Burke W (2001). HFE gene and hereditary hemochromatosis: a HuGE review. Am J Epidemiol.

[3] Finch CA, Hegsted M, Kinney TD (1950). Iron metabolism: the pathophysiology of iron storage. Blood.

[4] McLaren GD, Gordeuk VR (2009). Hereditary hemochromatosis: insights from the Hemochromatosis and Iron Overload Screening (HEIRS) Study. Hematology Am Soc Hematol Educ Program.

[5] Grosse SD, Gurrin LC, Bertalli NA, Allen KJ (2018). Clinical penetrance in hereditary hemochromatosis: estimates of the cumulative incidence of severe liver disease among HFE C282Y homozygotes. Genet Med.

[6] Koshy A, Mukkada RJ, Chettupuzha AP, Francis JV, Kandathil JC, Mahadevan P (2020). Hemochromatosis in India: first report of whole exome sequencing with review of the literature. J Clin Exp Hepatol.

[7] Garewal G, Das R, Ahluwalia J, Marwaha RK (2005). Prevalence of the H63D mutation of the HFE in north India: its presence does not cause iron overload in beta thalassemia trait. Eur J Haematol.

[8] Matissek SJ, Elsawa SF (2020). GLI3: a mediator of genetic diseases, development and cancer. Cell Commun Signal.

[9] Gochee PA, Powell LW, Cullen DJ, Du Sart D, Rossi E, Olynyk JK (2002). A population-based study of the biochemical and clinical expression of the H63D hemochromatosis mutation. Gastroenterology.

[10] Girelli D, Marchi G, Busti F (2024). Diagnosis and management of hereditary hemochromatosis: lifestyle modification, phlebotomy, and blood donation. Hematology Am Soc Hematol Educ Program.

[11] Savatt JM, Johns A, Schwartz ML (2023). Testing and management of iron overload after genetic screening-identified hemochromatosis. JAMA Netw Open.

